# Fulminant Klebsiella pneumoniae Endogenous Endophthalmitis With Globe Perforation and Disseminated Infection: A Case Report

**DOI:** 10.7759/cureus.114467

**Published:** 2026-08-13

**Authors:** Robert M Branstetter, Landon T Godso, Wayne J Wortmann, Christine W Connolly

**Affiliations:** 1 Department of Ophthalmology, Louisiana State University Health Sciences Center, New Orleans, USA

**Keywords:** cerebral septic emboli, endogenous klebsiella endophthalmitis, hepatic vein thrombosis, oculoplastic surgeries, ophthalmology case report, pneumonia, septic emboli, surgical enucleation

## Abstract

Endogenous endophthalmitis (EE) occurs when microorganisms spread hematogenously to the eye, resulting in intraocular infection. While Gram-positive organisms are the most common causes in the United States, hypervirulent *Klebsiella pneumoniae* has emerged as an increasingly recognized pathogen associated with liver abscess and metastatic infection. We report the case of a 51-year-old woman with previously undiagnosed diabetes who presented with progressive vision loss in the right eye and systemic symptoms after recent travel to China. Ophthalmic examination demonstrated hypopyon and severe intraocular inflammation consistent with endogenous endophthalmitis. Blood and vitreous cultures grew *Klebsiella pneumoniae*. Despite prompt treatment with intravitreal and systemic antibiotics, the patient developed extensive disseminated infection, including multifocal pneumonia, cerebral septic emboli, and a large multiloculated liver abscess with hepatic venous thrombosis requiring drainage. The affected eye rapidly progressed to no light perception with development of a scleral abscess and spontaneous globe perforation, ultimately necessitating enucleation. This case highlights the aggressive metastatic potential of hypervirulent *K. pneumoniae* infection and underscores the importance of early recognition, systemic evaluation for occult abscesses, and coordinated multidisciplinary management. Increasing reports of *Klebsiella* endogenous endophthalmitis in the United States suggest expanding global dissemination of this pathogen.

## Introduction

Endogenous endophthalmitis (EE) arises when microorganisms spread from a hematogenous source, leading to intraocular infection [1]. In the United States, Gram-positive organisms such as *Streptococcus* species and *Staphylococcus aureus* are most commonly implicated [2,3]. In contrast, *Klebsiella pneumoniae* is the most frequent cause of EE in parts of Asia, with reported rates of up to 1% in New Caledonia and 3.8% in Singapore of patients with *K. pneumoniae* bacteremia [4-6]. Hypervirulent and multidrug-resistant strains of *K. pneumoniae* have now been documented in multiple regions worldwide, including the United States, Australia, Europe, and Africa, demonstrating increased global spread. Approximately 90% of these cases present with concurrent liver abscesses, and the source of infection appears to be gastrointestinal, oropharyngeal, or urinary in origin [7]. A retrospective Korean study of 97 eyes identified several major risk factors for *Klebsiella* EE in their cohort, including concurrent liver abscess (25%), diabetes (42.5%), and liver cirrhosis (20%) [8].

Although early diagnosis and treatment with pars plana vitrectomy, intravitreal antibiotic injection, and abscess drainage may help with preservation of initial visual acuity on presentation, overall visual prognosis remains poor [3,9]. Studies have demonstrated that more than 75% of patients with *K. pneumoniae* endophthalmitis progress to only hand-motion vision or require evisceration or enucleation, even with prompt intervention [10,11].

In this report, we present the case of a 51-year-old woman with previously undiagnosed diabetes who developed *Klebsiella pneumoniae* EE following recent travel to China. Her clinical course was complicated by multifocal pneumonia, liver abscess with hepatic venous thrombosis, cerebral septic emboli, and ultimately enucleation of the affected eye.

## Case presentation

A 51-year-old woman with a medical history of hypertension and tobacco use presented to an outside emergency department (ED) after five days of progressive blurry vision in the right eye, complete vision loss over the preceding two days, and associated chest pain. She also reported fatigue, generalized weakness, fever for two days, and a nonproductive cough that worsened when supine. Notably, she had recently traveled to urban areas in China three months prior. She denied trauma, past ocular disease, abdominal pain, headache, or visual changes in the left eye.

On presentation to an outside ED, her fever, tachycardia, tachypnea, and leukocytosis with bandemia prompted concern for sepsis. Her temperature was 100.3°F, heart rate was 115 beats per minute, and respirations were 27 per minute. Laboratory evaluation (Table 1) demonstrated leukocytosis (26.2 × 10³/µL with 14% bandemia), metabolic derangements, hyperglycemia (317 mg/dL), elevated alkaline phosphatase (216 U/L), and a procalcitonin level of 7.63 ng/mL. Hemoglobin A1c was 13%, consistent with previously undiagnosed diabetes mellitus. Chest radiograph showed a left upper lobe opacity, and computed tomography (CT) of the chest demonstrated marked consolidation of the left upper lobe with additional scattered nodular opacities bilaterally, concerning for multifocal pneumonia. CT of the orbits was unremarkable apart from mild symmetric proptosis. She was treated with intravenous cefepime and vancomycin for suspected pneumonia and the hypopyon, intravenous fluids, and insulin, before being transferred to a tertiary care center for further management.

**Table 1:** Laboratory Findings at Initial Presentation Initial laboratory findings on presentation to the emergency department AST: aspartate aminotransferase, ALT: alanine aminotransferase

Laboratory Test	Patient Value	Reference Range
White Blood Cell Count (×10³/µL)	26.2	4.0-11.0
Band Neutrophils (%)	14	0-5
Sodium (mmol/L)	130	136-145
Potassium (mmol/L)	2.8	3.5-5.1
Chloride (mmol/L)	96	98-107
Carbon Dioxide (CO₂) (mmol/L)	17	22-29
Anion Gap (mmol/L)	17	7-16
Calcium (mg/dL)	7.9	8.6-10.2
Blood Urea Nitrogen (mg/dL)	12	6-20
Creatinine (mg/dL)	0.28	0.51-0.95
Glucose (mg/dL)	317	70-99
Beta-Hydroxybutyrate (mmol/L)	5.2	<0.6
AST (U/L)	16	10-40
ALT (U/L)	25	7-56
Alkaline Phosphatase (U/L)	216	44-147
Albumin (g/dL)	2.7	3.5-5.0
Procalcitonin (ng/mL)	7.63	<0.10
Hemoglobin A1c (%)	13	<5.7

She arrived via ground transport to our hospital the following day, and ophthalmology was consulted for hypopyon of the right eye as well as her other aforementioned visual symptoms. Visual acuity was light perception in the right eye (OD) and 20/20 in the left (OS). Intraocular pressures (IOP) were 9 mmHg OD and 10 mmHg OS. The right pupil was nonreactive. Examination revealed diffuse conjunctival injection and chemosis, a layered hypopyon with fibrin in the anterior chamber, posterior synechiae, and an anterior fibrinous membrane obscuring view to the posterior segment in the right eye. The left eye was normal. Endogenous endophthalmitis was suspected, and the patient underwent vitreous biopsy with intravitreal vancomycin (1 mg/0.1 mL) and ceftazidime (2.25 mg/0.1 mL). Vitreous samples were sent for aerobic and anaerobic bacterial culture, Gram stain, and fungal studies. Empiric antimicrobial therapy was initiated with piperacillin-tazobactam (4.5 g intravenously every 8 hours) and azithromycin (500 mg orally every 24 hours). Vancomycin was withheld because methicillin-resistant *Staphylococcus aureus* (MRSA) polymerase chain reaction (PCR) testing performed at the outside hospital was negative.

**Figure 1:**
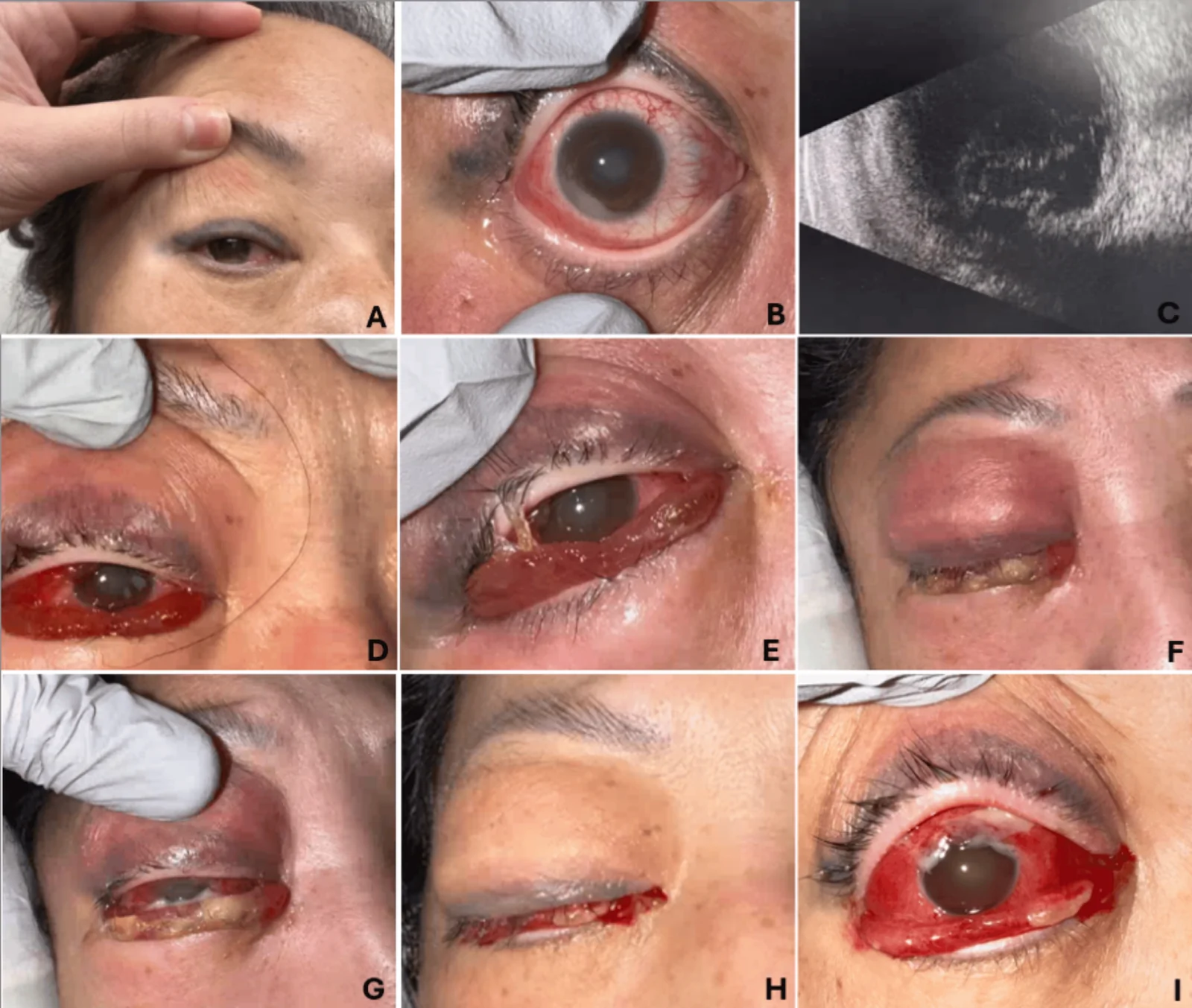
Clinical Progression of Klebsiella pneumoniae Endogenous Endophthalmitis (A) Day prior to admission: Conjunctival injection and inferior hypopyon of the right eye. (B) Hospital day 1: Diffuse conjunctival injection and chemosis, an inferior layered hypopyon with fibrin within the anterior chamber, posterior synechiae, and a fibrinous anterior membrane obscuring the posterior segment. (C) Hospital day 3: Ophthalmic ultrasound (B-scan) revealed diffuse choroidal thickening with intraocular abscesses. (D and E) Hospital day 5: Worsening eyelid edema, conjunctival chemosis, and diffuse inflammation. (F and G) Hospital day 9: External purulent discharge noted with worsening eyelid edema, conjunctival chemosis, and diffuse inflammation. (H and I) Hospital day 12: Sanguineous discharge, subconjunctival hemorrhage, and a new scleral abscess adjacent to the superior limbus.

Over the following days, her ocular status failed to improve. Figure 1 demonstrates serial clinical photographs of the right eye from hospital day 1 through day 12. On hospital day 2, she was without light perception in the right eye, with elevated intraocular pressure to 23 mmHg. Her antimicrobial regimen was tailored to piperacillin-tazobactam (4.5 g intravenously every 8 hours) and levofloxacin (750 mg orally every 24 hours), with levofloxacin selected for its superior ocular penetration. The next day, examination showed worsening eyelid edema, conjunctival chemosis, and diffuse inflammation. Ophthalmic ultrasound (B-scan) revealed diffuse choroidal thickening with intraocular abscesses. Vitreous and blood cultures both grew Gram-negative rods later identified as *Klebsiella pneumoniae, *susceptible to cefazolin, ciprofloxacin, gentamicin, and trimethoprim/sulfamethoxazole*. *Antimicrobial therapy was changed to ceftriaxone (2 g intravenously every 12 hours) and levofloxacin (750 mg orally every 24 hours) based on culture and susceptibility results identifying *Klebsiella pneumoniae.*

Intraocular pressure continued to increase to 32 mmHg with worsening inflammation on hospital day 4. Given the degree of eye inflammation, chemosis, and elevated IOP, we recommended further imaging to look for possible venous sinus thrombosis or orbital space-occupying lesion. Magnetic resonance imaging (MRI) of the orbits was thus obtained and revealed diffuse orbital inflammatory changes consistent with endophthalmitis, as well as multiple microabscesses scattered throughout both cerebral hemispheres, consistent with septic emboli. No cavernous sinus thrombosis or drainable orbital collection was identified. A transthoracic echocardiogram performed earlier showed no valvular vegetations or thrombus. Topical brimonidine and dorzolamide were initiated for comfort.

Over the next several days, her right eye remained without light perception, but intraocular surgery was postponed due to concern for her ability to handle general anesthesia and the possibility of starting her on anticoagulants. Approximately one week after admission, CT of the abdomen/pelvis was obtained due to positive *K. pneumoniae* cultures, which revealed a 6.7 × 5.6 × 5.5 cm multiloculated liver abscess with hepatic venous thrombosis extending into the right hepatic vein, numerous cavitary and necrotic pulmonary nodules consistent with septic emboli, and a small pulmonary infarct distal to a macroscopic embolus in the right lower lobe. Interventional radiology drained the hepatic abscess, and neurosurgery was consulted regarding the safety of therapeutic anticoagulation in the setting of concurrent cerebral septic emboli.

Despite systemic management, the right eye continued to worsen. On hospital day 9, purulent discharge was noted externally, although the orbit appeared less tense. Enucleation versus incision and drainage (I&D) was discussed with the patient. By hospital day 11, a new scleral abscess adjacent to the superior limbus had developed. The infectious disease service recommended continuing ceftriaxone (2 g intravenously every 12 hours) and levofloxacin (750 mg orally every 24 hours) and adding metronidazole (500 mg orally every 8 hours). Although enucleation was the preferred definitive treatment given the patient's no-light-perception vision, persistent infection, and severe pain, she was not initially medically cleared for general anesthesia because of her poor overall clinical status. In addition, definitive surgical management was deferred while the multidisciplinary team determined whether therapeutic anticoagulation would be required for the patient's hepatic vein thrombosis and cerebral septic emboli. Although neurosurgery ultimately cleared the patient for therapeutic anticoagulation, the gastroenterology team recommended against initiating anticoagulation beyond prophylactic-dose heparin for deep vein thrombosis prophylaxis because her liver enzyme levels remained within normal limits. As enucleation could not yet be safely performed, I&D was scheduled as a temporizing measure. However, the patient's clinical status improved before the procedure, and once she was medically optimized and cleared for general anesthesia on hospital day 12, enucleation was performed in lieu of I&D.

**Figure 2:**
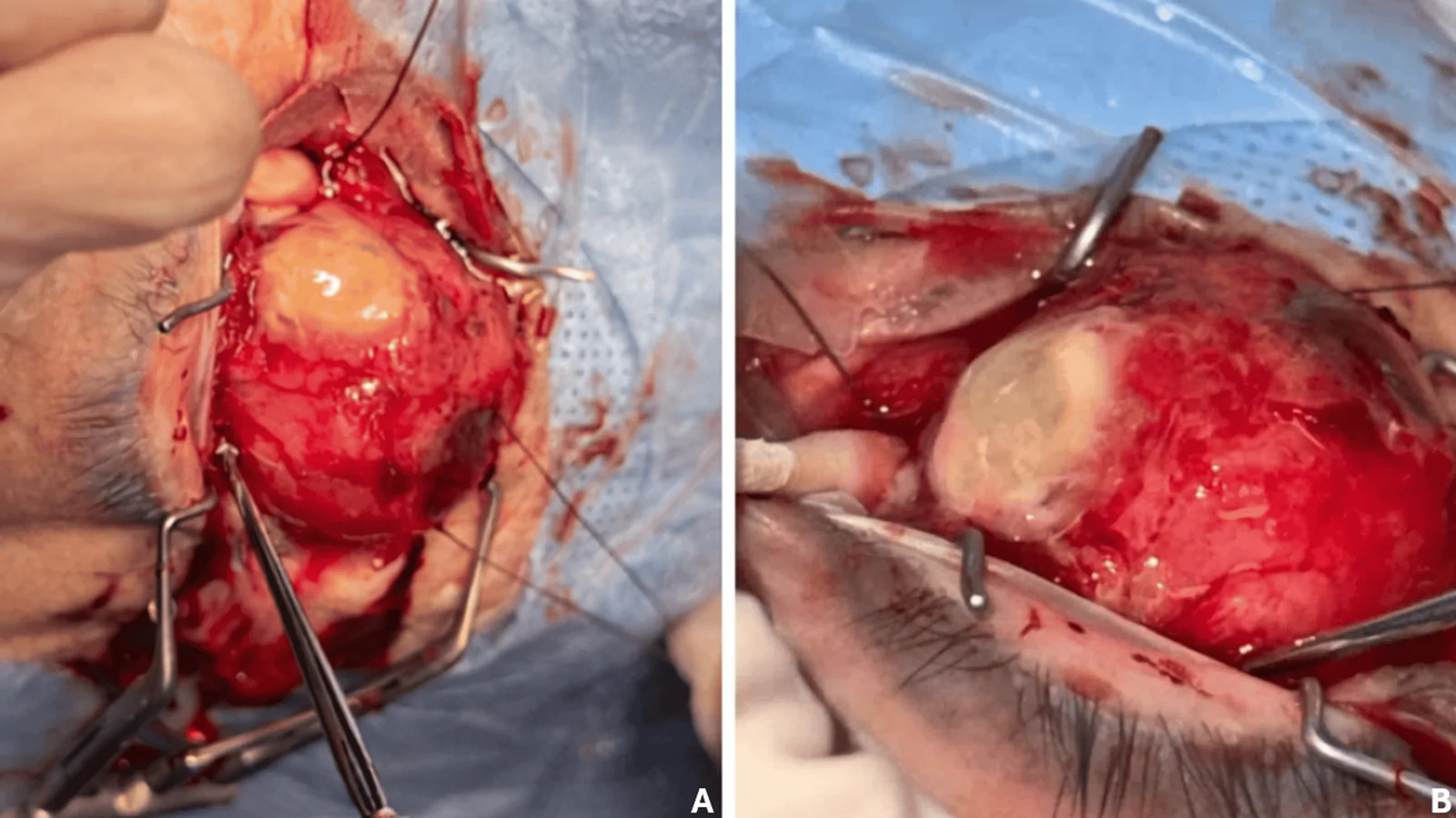
Superior Scleral Abscess With Spontaneous Globe Perforation (A) Anterior intraoperative view demonstrating a large scleral abscess with associated scleral thinning and necrosis. (B) Oblique intraoperative view demonstrating the extent of the elevated scleral abscess and surrounding inflammatory changes.

Intraoperative exploration revealed a scleral abscess with spontaneous globe perforation (Figure 2). Given the unsalvageable nature of the eye, enucleation was performed. Operative findings included extensive 360-degree Tenon's adhesions, a large superior scleral abscess with purulent efflux, and absence of the superior rectus and superior oblique muscles, which were suspected to be eroded by the infection. Purulent material and the globe were sent for microbiologic and pathologic analysis. Due to active infection, no orbital implant was placed. The orbit was irrigated with gentamicin, the conjunctiva and Tenon's capsule were closed, and a conformer was placed. A temporary tarsorrhaphy was performed.

Postoperatively, the patient's orbital pain improved significantly, and there was no further purulent drainage. She was counseled on conformer care and planned for tarsorrhaphy removal one week later. Levofloxacin was discontinued because enhanced ocular penetration was no longer necessary after removal of the infected globe. The patient remained on ceftriaxone and metronidazole to complete treatment for invasive *Klebsiella pneumoniae* syndrome. Final histopathologic examination demonstrated an orbital abscess with the vitreous cavity entirely replaced by amorphous debris and acute inflammatory cells. Four days after surgery, follow-up CT of the chest revealed a right pleural effusion and persistent partially cavitated consolidation of the left upper lobe, for which pulmonology was consulted. The patient remained hospitalized because of her evolving disseminated infection and the need for prolonged intravenous antimicrobial therapy. Approximately six weeks later, the lateral tarsorrhaphy was removed. On hospital day 53, despite extensive counseling regarding the risks of premature discontinuation of treatment, the patient elected to leave the hospital against medical advice. She was therefore discharged on indefinite oral levofloxacin suppressive therapy. Following discharge, the patient returned to China to seek a second opinion and was subsequently lost to follow-up; therefore, no additional clinical outcome data were available.

## Discussion

Epidemiology

*Klebsiella pneumoniae* endogenous endophthalmitis (EE) has historically been concentrated in Asian regions but has become increasingly recognized worldwide [7]. Reports of *K. pneumoniae* EE in the United States have been rare, with only three cases published prior to 2000, including two in Florida in 1994 and one in Arkansas in 1999 [12,13]. Recognition increased in the following decade, with two additional cases reported in a 2013 review [14] and a 2014 case series describing three cases of* K. pneumoniae* EE [15]. More recently, larger series and individual reports have further highlighted its emergence. A 2019 retrospective review from Stanford and Santa Clara Valley Medical Center identified 10 patients (12 eyes) diagnosed between 2000 and 2017 [16]. In Los Angeles, a 2021 case report described a diabetic patient with no travel history and local infection by a genetically characterized hypervirulent* K. pneumoniae* (hvKp) strain [17]. Hypervirulent *K. pneumoniae* is distinguished from classical strains by enhanced pathogenic features that promote invasive disease and hematogenous spread, including mechanisms that increase iron acquisition and capsular production [17]. A subsequent 2025 series from the same region described four additional cases of *Klebsiella* EE [3]. Collectively, these reports demonstrate increasing recognition of* K. pneumoniae* EE in the United States, although the absence of molecular characterization in many reported cases precludes attributing this geographic trend specifically to confirmed hvKp strains.

Current case comparison and significance

We report this patient because the existing literature rarely describes such extensive ocular destruction and multifocal systemic infection from *K. pneumoniae* EE within the United States. To our knowledge, this is the third case of endogenous *K. pneumoniae* endophthalmitis reported in New Orleans, Louisiana. Two prior cases of *K. pneumoniae* EE were reported from Tulane University in New Orleans. Both similarly involved liver abscess-associated endogenous infection and resulted in enucleation despite systemic therapy. Dodson et al. described a 51-year-old man with positive cultures from the urine, blood, liver, and vitreous [18], while Victor et al. reported rapid progression despite abscess drainage and systemic antibiotics [19]. The present case was notable for an unusually destructive ocular course, including scleral abscess formation, spontaneous globe perforation, and involvement of the extraocular musculature, accompanied by extensive systemic dissemination with pulmonary and cerebral septic emboli. This combination of severe ocular destruction and multisystem metastatic infection highlights the particularly aggressive presentation observed in our patient. Additionally, previous literature has reported concurrent liver abscesses in approximately 90% of cases of* K. pneumoniae* EE [7], emphasizing the importance of abdominal imaging when endogenous *K. pneumoniae* infection is suspected.

Unique clinical findings in this case

Intraoperative findings in our patient were distinctive, including a scleral abscess with spontaneous globe perforation and near-total loss of the superior rectus and superior oblique muscles, demonstrating the locally destructive course of infection. Although severe ocular extension with scleral necrosis and perforation has been described in prior *K. pneumoniae* EE [20], the extensive extraocular muscle destruction observed in our patient further demonstrates the locally invasive nature of the infection. Systemic dissemination was similarly extensive, including cerebral septic emboli, hepatic vein thrombosis, pulmonary emboli, and cavitary pulmonary nodules. Although septic embolization and metastatic involvement of multiple organ systems have been described in prior reports [21-23], the combination of extensive multisystem dissemination with scleral abscess formation, extraocular muscle destruction, and spontaneous globe perforation represents an unusually aggressive clinical presentation. This severity may have been exacerbated by impaired host immunity secondary to uncontrolled diabetes. Our patient's previously undiagnosed diabetes aligns with existing literature identifying diabetes as a risk factor for* K. pneumoniae* EE, with one Korean case series reporting diabetes in 42.5% of affected patients [8]. However, the severity and extent of disseminated infection are likely multifactorial and cannot be attributed to diabetes alone. Although molecular characterization of the isolate was not performed in our patient, the presence of a liver abscess with bacteremia and metastatic infection involving the eye, lungs, and central nervous system was clinically suggestive of the invasive phenotype associated with hypervirulent *K. pneumoniae*. Accordingly, hypervirulence in this case was inferred from the clinical pattern of disseminated infection rather than confirmed by molecular characterization.

Visual prognosis and treatment considerations

In our case, intravitreal vancomycin and ceftazidime, along with systemic antibiotics, were initiated on the day of admission, approximately five days after symptom onset, yet the eye rapidly deteriorated to no light perception. Despite prompt treatment upon presentation, over 75% of patients with* K. pneumoniae* endophthalmitis experience profound visual loss, often limited to hand motion or worse, and many ultimately require evisceration or enucleation [10,11]. Early recognition remains critical, as prompt intravitreal antimicrobial therapy, pars plana vitrectomy in appropriately selected patients, and drainage of the infectious source have been associated with improved visual outcomes and a greater likelihood of globe preservation [3,9].

Management of endogenous *K. pneumoniae *endophthalmitis requires prompt systemic antimicrobial therapy, intravitreal antibiotics, and definitive source control. Although pars plana vitrectomy may improve outcomes in selected eyes with salvageable vision, advanced infection characterized by no-light-perception vision, extensive scleral or orbital involvement, globe perforation, or progression despite aggressive medical therapy may preclude meaningful globe salvage and necessitate enucleation or evisceration. In the present case, definitive surgical management was delayed because the patient was not medically stable enough to undergo general anesthesia and required multidisciplinary evaluation regarding potential therapeutic anticoagulation rather than because of uncertainty regarding ophthalmic management. Although the extent to which this delay contributed to the final ocular outcome cannot be determined, the patient had already presented with advanced infection and subsequently progressed to no-light-perception vision. Given her delayed presentation, no-light-perception vision, scleral abscess with globe perforation, and disseminated invasive infection, globe salvage was considered unlikely despite prompt intravitreal and systemic antimicrobial therapy once she presented for care.

## Conclusions

This case underscores the fulminant course and destructive potential of *Klebsiella pneumoniae* endogenous endophthalmitis. The combination of extraocular muscle destruction, scleral abscess formation, spontaneous globe perforation, and widespread septic embolization highlights the unusually aggressive ocular and systemic manifestations observed in this patient. Although molecular characterization was not performed, the extensive pattern of metastatic infection was clinically suggestive of the invasive phenotype associated with hypervirulent* K. pneumoniae.* This case reinforces the importance of early recognition, targeted antimicrobial therapy, source control, and coordinated ophthalmic and systemic management in patients with disseminated *K. pneumoniae* infection.
